# Registry-defined incident chronic kidney disease following acute kidney injury after primary PCI for STEMI and its association with long-term outcomes: a multicenter landmark analysis

**DOI:** 10.3389/fcvm.2026.1933650

**Published:** 2026-10-27

**Authors:** Yong-Lee Kim, Jeong Yoon Jang, Jae Seok Bae, Yun-Ho Cho, Yujin Shin, Han Ra Choi, Hyeong Joo Lee, Min Seok Noh, Jeong Rang Park, Min Gyu Kang, Hye-Ree Kim, Hyo Jin Lee, Kye-Hwan Kim, Jin-Yong Hwang, Sung-Eun Park, Jong-Hwa Ahn

**Affiliations:** 1 Division of Cardiology, Department of Internal Medicine, Gyeongsang National University School of Medicine and Gyeongsang National University Changwon Hospital, Changwon, Republic of Korea; 2 Department of Internal Medicine, Gyeongsang National University School of Medicine and Gyeongsang National University Hospital, Jinju, Republic of Korea; 3 Department of Radiology, Gyeongsang National University School of Medicine and Gyeongsang National University Changwon Hospital, Changwon, Republic of Korea

**Keywords:** acute kidney injury, cardiorenal syndrome, chronic kidney disease, landmark analysis, percutaneous coronary intervention, ST-elevation myocardial infarction

## Abstract

**Background:**

Acute kidney injury (AKI) frequently occurs in patients with ST-segment elevation myocardial infarction (STEMI) undergoing primary percutaneous coronary intervention (PCI). Kidney recovery after AKI is heterogeneous, and the prognostic significance of kidney dysfunction persisting to the 3−6-month assessment remains incompletely defined. We evaluated whether registry-defined incident chronic kidney disease (CKD) following AKI was associated with subsequent death and myocardial infarction (MI).

**Methods:**

This retrospective analysis of a prospectively collected multicenter PCI registry included STEMI patients treated with primary PCI between January 2010 and November 2020. AKI was defined according to KDIGO criteria. Kidney trajectory was classified as no AKI, transient AKI with recovery, or registry-defined incident CKD following AKI based on kidney function assessed at 3–6 months. To minimize guarantee-time bias, Kaplan–Meier and Cox analyses began at a 6-month landmark. Multivariable Cox models adjusted for age, sex, diabetes mellitus, LVEF, and baseline MDRD eGFR.

**Results:**

Among 1,239 patients, 941 had no AKI, 56 had transient AKI, and 242 had incident CKD following AKI; the overall AKI incidence was 24.1%. The strict 6-month landmark cohort comprised 1,129 patients (869 no AKI, 48 transient AKI, and 212 incident CKD). Compared with no AKI, incident CKD was associated with all-cause death (multivariable Cox HR 4.06, 95% CI 1.23–13.45; *P* = 0.022), MI (multivariable Cox HR 4.49, 95% CI 1.79−11.30; *P* = 0.001), and death and/or MI (multivariable Cox HR 4.35, 95% CI 2.07−9.14; *P* < 0.001), whereas transient AKI was not significantly associated with these outcomes. In the two-group analysis, incident CKD was similarly associated with death (multivariable Cox HR 3.44, 95% CI 1.18−10.06; *P* = 0.024), MI (multivariable Cox HR 3.75, 95% CI 1.59−8.87; *P* = 0.003), and death and/or MI (multivariable Cox HR 3.61, 95% CI 1.83−7.14; *P* < 0.001).

**Conclusions:**

Registry-defined incident CKD after in-hospital AKI identified a subgroup of STEMI survivors with substantially higher post-landmark risks of death and MI. Kidney recovery trajectory may provide clinically useful prognostic information beyond the occurrence of AKI alone.

**Trial registration:**

ClinicalTrials.gov identifier: NCT04650529. Registered on 25 January 2021. Retrospectively registered.

## Introduction

nAcute kidney injury (AKI) is a frequent complication in patients with ST-segment elevation myocardial infarction (STEMI) undergoing primary percutaneous coronary intervention (PCI) and is consistently associated with adverse short- and long-term outcomes (1–4). The mechanisms of AKI in this setting are multifactorial and include kidney hypoperfusion caused by hemodynamic instability, systemic inflammation, neurohormonal activation, and exposure to iodinated contrast media during PCI (4).

AKI is increasingly recognized as more than a transient laboratory abnormality. Maladaptive repair after an acute kidney insult may lead to persistent inflammation, nephron loss, tubulointerstitial fibrosis, and subsequent chronic kidney disease (CKD) (5, 6). Importantly, kidney recovery after AKI is heterogeneous. Prior STEMI studies have shown that delayed or incomplete kidney recovery after AKI is common and carries adverse prognostic implications (7–9). Kofman et al. reported worse long-term kidney outcomes and mortality among STEMI patients with prolonged kidney dysfunction after primary PCI, whereas Itach et al. demonstrated that delayed or absent AKI recovery was associated with markedly worse long-term survival. More recently, Frydman et al. showed that acute kidney disease persisting for 7–90 days after STEMI-associated AKI was common and associated with higher 1-year mortality (7–9).

Most prior kidney-trajectory studies in STEMI have focused on recovery during hospitalization or within the acute kidney disease period of up to 90 days. Less is known about the prognostic significance of kidney dysfunction still present at the later 3–6-month assessment, particularly among STEMI patients without recognized baseline CKD. Moreover, studies that classify kidney status months after the index STEMI require careful handling of guarantee-time bias because patients must survive and remain under observation long enough for kidney trajectory to be ascertained.

Accordingly, the present study evaluated kidney trajectories after primary PCI for STEMI and their associations with subsequent all-cause death and myocardial infarction (MI) using a 6-month landmark design. We hypothesized that registry-defined incident CKD following AKI, rather than transient AKI with kidney recovery, would identify patients at particularly high long-term cardiovascular risk.

## Methods

### Study population

This study was a retrospective analysis of prospectively collected data from a multicenter PCI registry involving Gyeongsang National University Changwon Hospital and Gyeongsang National University Hospital (ClinicalTrials.gov identifier: NCT04650529) (10). Consecutive patients with coronary artery disease undergoing PCI between January 2010 and November 2020 were prospectively registered using standardized institutional protocols. Baseline demographic, clinical, laboratory, and procedural variables were collected within the registry, while post-discharge kidney function and long-term clinical outcomes were ascertained from registry data and institutional electronic medical records.

For the present analysis, 1,486 patients presenting with STEMI who underwent primary PCI were identified. Patients with baseline CKD were excluded to evaluate *de novo* post-AKI kidney dysfunction. Baseline CKD was defined operationally as a documented history of CKD before the index hospitalization or kidney function below the registry CKD threshold at admission. Albuminuria and urine albumin-to-creatinine ratio were not systematically available and therefore were not used to identify baseline CKD with preserved kidney function. After exclusion of 247 patients with baseline CKD, 1,239 patients constituted the kidney-trajectory cohort (Figure 1). An available kidney function measurement within the 3–6-month assessment window was required for trajectory classification, and all 1,239 patients included in the kidney-trajectory cohort had such a measurement (100%).

**Figure 1:**
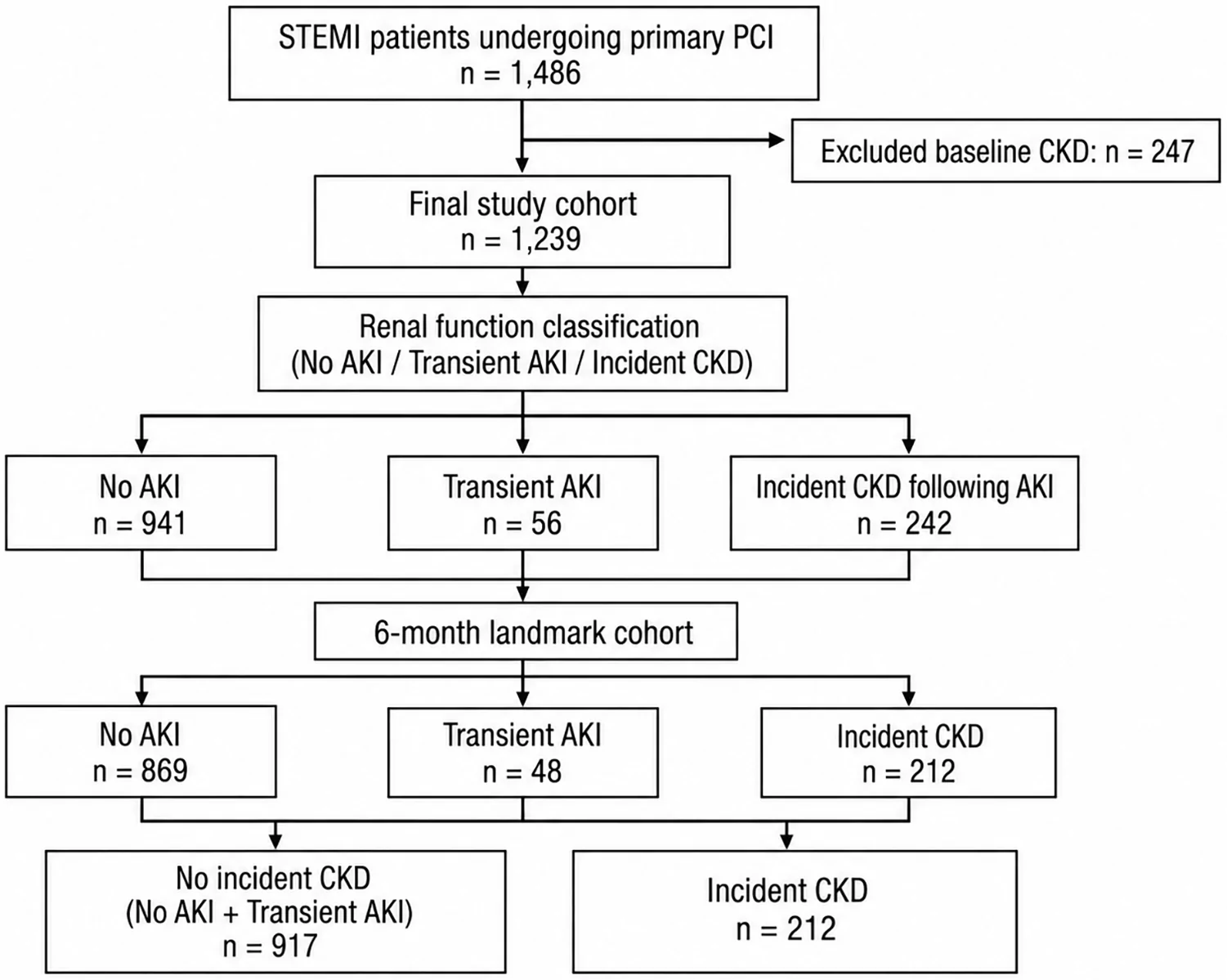
Study flow diagram. Of 1,486 STEMI patients undergoing primary PCI, 247 with baseline CKD were excluded, leaving 1,239 patients in the kidney-trajectory cohort: no AKI (*n* = 941), transient AKI (*n* = 56), and registry-defined incident CKD following AKI (*n* = 242). The strict 6-month landmark cohort comprised 1,129 patients: no AKI (*n* = 869), transient AKI (*n* = 48), and incident CKD (*n* = 212). For the complementary two-group analysis, 917 patients were classified as no incident CKD and 212 as incident CKD. AKI, acute kidney injury; CKD, chronic kidney disease; PCI, percutaneous coronary intervention; STEMI, ST-segment elevation myocardial infarction.

### Definition of acute kidney injury

AKI was diagnosed according to Kidney Disease: Improving Global Outcomes (KDIGO) criteria: (1) an increase in serum creatinine of at least 0.3 mg/dL (approximately 26.5 µmol/L) within 48 h or (2) an increase in serum creatinine to at least 1.5 times the baseline value within 7 days (11). Serum creatinine was measured at admission and serially during hospitalization.

### Definition of kidney trajectory and registry-defined incident CKD

The original registry kidney classification used creatinine clearance estimated by the Cockcroft-Gault equation (12). Registry-defined incident CKD required (1) no recognized baseline CKD, (2) in-hospital AKI, and (3) Cockcroft-Gault-estimated creatinine clearance <60 mL/min at the available 3–6-month follow-up assessment. Transient AKI was defined as in-hospital AKI followed by recovery above this registry threshold at the 3–6-month assessment. The kidney-trajectory classification was based on the scheduled follow-up measurement available within this window; serial post-discharge measurements sufficient to document chronicity by contemporary KDIGO criteria were not systematically available for every patient. Because Cockcroft-Gault estimates creatinine clearance rather than body-surface-area-normalized eGFR, and because chronicity was not confirmed by repeated measurements in every patient, we use the term registry-defined incident CKD as an operational phenotype and acknowledge these limitations. MDRD eGFR values are additionally reported descriptively in Tables 1, 3.

**Table 1:** Baseline characteristics according to kidney trajectory.

Variable	No AKI (*n* = 941)	Transient AKI (*n* = 56)	Incident CKD following AKI (*n* = 242)	*P* value
Demographics/risk factors				
Age, years	59.0 [52.0–66.0]	75.0 [68.8–79.0]	79.0 [74.0–83.8]	<0.001
Male sex	809 (86.0%)	37 (66.1%)	114 (47.1%)	<0.001
Hypertension	369 (39.2%)	25 (44.6%)	129 (53.3%)	<0.001
Diabetes mellitus	170 (18.1%)	12 (21.4%)	53 (21.9%)	0.355
Dyslipidemia	644 (68.4%)	29 (51.8%)	121 (50.0%)	<0.001
Current smoker	510 (54.2%)	18 (32.1%)	63 (26.0%)	<0.001
Previous MI	37 (3.9%)	2 (3.6%)	11 (4.5%)	0.896
Previous PCI	69 (7.3%)	9 (16.1%)	22 (9.1%)	0.053
Previous CABG	4 (0.4%)	0 (0.0%)	1 (0.4%)	0.888
Previous stroke	30 (3.2%)	4 (7.1%)	22 (9.1%)	<0.001
Atrial fibrillation	19 (2.0%)	1 (1.8%)	1 (0.4%)	0.225
Presentation/procedure				
SBP at admission, mmHg	130.0 [115.0–149.0]	115.5 [100.0–130.2]	130.0 [110.0–145.8]	<0.001
DBP at admission, mmHg	80.0 [70.0–90.0]	70.0 [60.0–80.0]	75.0 [65.2–90.0]	<0.001
Heart rate at admission, bpm	76.0 [66.0–86.0]	75.5 [56.8–85.8]	72.0 [61.0–84.0]	0.029
LVEF, %	55.0 [50.0–60.0]	55.0 [48.8–61.2]	52.0 [45.0–59.0]	<0.001
Complex PCI	133 (14.1%)	7 (12.5%)	33 (13.6%)	0.930
Multivessel PCI	90 (9.6%)	6 (10.7%)	23 (9.5%)	0.959
Left main PCI	10 (1.1%)	0 (0.0%)	3 (1.2%)	0.712
PCI failure	6 (0.7%)	0 (0.0%)	1 (0.4%)	0.764
Kidney function				
Baseline creatinine, mg/dL	0.9 [0.8–1.0]	0.9 [0.8–1.1]	0.9 [0.8–1.0]	0.004
Baseline eGFR (MDRD), mL/min/1.73 m^2^	93.0 [80.9–106.0]	71.9 [63.2–85.0]	75.7 [67.8–87.0]	<0.001
3–6-month creatinine, mg/dL	0.9 [0.8–1.0]	0.7 [0.6–0.9]	0.9 [0.8–1.1]	<0.001
3–6-month eGFR (MDRD), mL/min/1.73 m^2^	92.9 [81.5–105.1]	98.9 [84.7–116.4]	70.7 [62.0–81.9]	<0.001
Laboratory findings				
Total cholesterol, mg/dL	195.0 [165.0–222.0]	177.0 [144.5–209.2]	178.5 [146.2–214.0]	<0.001
Triglyceride, mg/dL	151.0 [106.0–220.0]	107.0 [68.5–175.5]	113.0 [81.0–158.0]	<0.001
HDL-C, mg/dL	41.0 [35.0–50.0]	44.0 [32.8–51.2]	45.0 [37.0–55.0]	<0.001
LDL-C, mg/dL	131.0 [102.0–154.0]	118.5 [89.8–147.5]	116.0 [90.0–147.0]	<0.001
NT-proBNP, pg/mL	130.1 [53.9–339.9]	314.6 [78.2–1,275.4]	588.7 [273.0–1,889.1]	<0.001
HbA1c, %	5.9 [5.6–6.5]	5.8 [5.6–6.0]	5.9 [5.6–6.4]	0.105
WBC, ×10^3^/µL	10.4 [8.2–12.7]	10.6 [7.7–13.7]	9.4 [7.6–11.8]	0.003
Hemoglobin, g/dL	14.6 [13.6–15.5]	13.4 [12.1–14.1]	12.7 [11.3–13.5]	<0.001
Platelet, ×10^3^/µL	242.0 [205.0–285.0]	255.0 [209.2–288.0]	240.0 [196.5–291.5]	0.446
Discharge medications				
Beta-blocker	769 (81.7%)	39 (69.6%)	181 (74.8%)	0.009
ACE inhibitor	118 (12.5%)	7 (12.5%)	28 (11.6%)	0.919
ARB	652 (69.3%)	37 (66.1%)	166 (68.6%)	0.870
Lipid-lowering therapy	901 (95.7%)	56 (100.0%)	231 (95.5%)	0.278
Aspirin	927 (98.5%)	54 (96.4%)	238 (98.3%)	0.485
Clopidogrel	684 (72.7%)	44 (78.6%)	208 (86.0%)	<0.001
Prasugrel	39 (4.1%)	4 (7.1%)	4 (1.7%)	0.079
Ticagrelor	191 (20.3%)	7 (12.5%)	24 (9.9%)	<0.001

Values are presented as median (interquartile range) or number (percentage). Comparisons among the three kidney-trajectory groups were performed using the Kruskal–Wallis test for continuous variables and the chi-square or Fisher exact test for categorical variables, as appropriate. Registry-defined incident CKD was based on the prespecified Cockcroft-Gault creatinine-clearance threshold at the 3–6-month assessment.

AKI, acute kidney injury; CKD, chronic kidney disease; eGFR, estimated glomerular filtration rate; PCI, percutaneous coronary intervention.

Patients were classified into three kidney trajectories: no AKI, transient AKI with kidney recovery by the 3–6-month assessment, and registry-defined incident CKD following AKI. For the complementary two-group analysis, the no-AKI and transient-AKI groups were combined as no incident CKD and compared with the incident-CKD group.

### Landmark design and clinical outcomes

Because kidney trajectory was determined using kidney function information obtained during the 3–6-month period after discharge, all time-to-event analyses were performed using a 6-month landmark to minimize guarantee-time (immortal-time) bias. Patients who experienced death or MI before 183 days or who were not under observation through the landmark did not contribute to the post-landmark outcome analyses. The strict 6-month landmark cohort comprised 1,129 patients: 869 with no AKI, 48 with transient AKI, and 212 with incident CKD (Figure 1). Follow-up time for Kaplan–Meier and Cox analyses began at the 6-month landmark, and only events occurring after the landmark were counted.

The clinical outcomes were all-cause death, MI, and the composite of death and/or MI, defined as the first occurrence of either component after the landmark. Clinical outcomes were ascertained through systematic review of registry data and medical records.

### Statistical analysis

Continuous variables are presented as median (interquartile range) and categorical variables as number (percentage). Comparisons among the three kidney-trajectory groups were performed using the Kruskal–Wallis test for continuous variables and the chi-square or Fisher exact test for categorical variables, as appropriate. For the two-group analysis, the Mann–Whitney U test was used for continuous variables and the chi-square or Fisher exact test for categorical variables.

Kaplan–Meier curves were constructed from the 6-month landmark and compared using the log-rank test. Cox proportional hazards regression was used to estimate hazard ratios (HRs) with 95% confidence intervals (CIs). In the three-group analysis, no AKI served as the reference group. In the two-group analysis, no incident CKD served as the reference group. Multivariable Cox models included age, sex, diabetes mellitus, left ventricular ejection fraction (LVEF), and baseline MDRD eGFR. These covariates were selected *a priori* as clinically relevant prognostic factors while limiting model complexity given the number of hard clinical events. Patients with missing covariates required for a given model were excluded from that model.

The proportional hazards assumption was evaluated using Schoenfeld residuals and inspection of residual patterns over time. Because evidence of time-dependent effects was present for some covariates, the Cox HRs are interpreted as average relative hazards over the observed post-landmark follow-up rather than as strictly constant effects. All statistical tests were two-sided, and a *P* value <0.05 was considered statistically significant.

## Results

### Study population and kidney trajectories

Of 1,486 STEMI patients undergoing primary PCI, 247 with baseline CKD were excluded, leaving 1,239 patients for kidney-trajectory classification (Figure 1). Among these, 941 (75.9%) had no AKI, 56 (4.5%) experienced transient AKI with kidney recovery, and 242 (19.5%) met the registry-defined incident CKD phenotype following AKI. Overall, 298 patients (24.1%) experienced in-hospital AKI. Baseline characteristics according to kidney trajectory are summarized in Table 1.

Patients who developed registry-defined incident CKD were substantially older, were less frequently male and current smokers, and had lower LVEF and hemoglobin concentrations and higher NT-proBNP levels than patients without AKI. Baseline and 3–6-month kidney-function measures also differed across the three trajectories. The transient-AKI group generally demonstrated an intermediate clinical phenotype.

### Clinical outcomes according to kidney trajectory

The strict 6-month landmark cohort included 1,129 patients: 869 with no AKI, 48 with transient AKI, and 212 with incident CKD. The median post-landmark follow-up duration was 4.23 years (interquartile range, 1.69–6.49 years). Post-landmark event rates were highest in the incident-CKD group (Table 2). All-cause death occurred in 13/869 (1.5%) patients with no AKI, 2/48 (4.2%) with transient AKI, and 18/212 (8.5%) with incident CKD. MI occurred in 30/869 (3.5%), 2/48 (4.2%), and 19/212 (9.0%), respectively. Death and/or MI occurred in 41/869 (4.7%), 4/48 (8.3%), and 35/212 (16.5%), respectively.

**Table 2:** Cox proportional hazards analyses according to kidney trajectory.

Outcome	Kidney trajectory	Events, n/N (%)	Unmultivariable Cox HR (95% CI)	*P* value	Multivariable Cox HR (95% CI)	*P* value
All-cause death	No AKI (reference)	13/869 (1.5%)	Reference	—	Reference	—
	Transient AKI	2/48 (4.2%)	3.56 (0.80–15.93)	0.096	2.95 (0.49–17.82)	0.238
	Incident CKD	18/212 (8.5%)	7.68 (3.71–15.91)	<0.001	4.06 (1.23–13.45)	0.022
Myocardial infarction	No AKI (reference)	30/869 (3.5%)	Reference	—	Reference	—
	Transient AKI	2/48 (4.2%)	1.50 (0.36–6.29)	0.579	1.51 (0.31–7.30)	0.608
	Incident CKD	19/212 (9.0%)	3.21 (1.80–5.72)	<0.001	4.49 (1.79–11.30)	0.001
Death and/or MI	No AKI (reference)	41/869 (4.7%)	Reference	—	Reference	—
	Transient AKI	4/48 (8.3%)	2.22 (0.79–6.22)	0.129	2.00 (0.62–6.44)	0.245
	Incident CKD	35/212 (16.5%)	4.51 (2.86–7.12)	<0.001	4.35 (2.07–9.14)	<0.001

Outcome analyses were performed in the strict 6-month landmark cohort (*n* = 1,129: no AKI *n* = 869, transient AKI *n* = 48, incident CKD *n* = 212). Follow-up began at the 6-month landmark. No AKI served as the reference group.

Adjusted models included age, sex, diabetes mellitus, LVEF, and baseline MDRD eGFR. HR, hazard ratio; CI, confidence interval; MI, myocardial infarction.

After multivariable adjustment, transient AKI was not significantly associated with all-cause death (multivariable Cox HR 2.95, 95% CI 0.49–17.82; *P* = 0.238), MI (multivariable Cox HR 1.51, 95% CI 0.31–7.30; *P* = 0.608), or death and/or MI (multivariable Cox HR 2.00, 95% CI 0.62–6.44; *P* = 0.245) compared with no AKI. In contrast, registry-defined incident CKD was associated with all-cause death (multivariable Cox HR 4.06, 95% CI 1.23–13.45; *P* = 0.022), MI (multivariable Cox HR 4.49, 95% CI 1.79–11.30; *P* = 0.001), and death and/or MI (multivariable Cox HR 4.35, 95% CI 2.07–9.14; *P* < 0.001).

### Two-group analysis according to incident CKD status

For the complementary two-group analysis, 917 patients without incident CKD (no AKI or transient AKI) were compared with 212 patients with registry-defined incident CKD in the landmark cohort. Their baseline characteristics are summarized in Table 3. Patients with incident CKD had higher cumulative post-landmark event rates for all three outcomes (Figure 2; all log-rank *P* < 0.001).

**Table 3:** Baseline characteristics according to incident CKD status in the 6-month landmark cohort.

Variable	No incident CKD (*n* = 917)	Incident CKD (*n* = 212)	*P* value
Demographics/risk factors			
Age, years	59.0 [52.0–67.0]	79.0 [74.0–83.0]	<0.001
Male sex	783 (85.4%)	102 (48.1%)	<0.001
Hypertension	362 (39.5%)	112 (52.8%)	<0.001
Diabetes mellitus	170 (18.5%)	46 (21.7%)	0.288
Dyslipidemia	623 (67.9%)	110 (51.9%)	<0.001
Current smoker	494 (53.9%)	59 (27.8%)	<0.001
Previous MI	36 (3.9%)	10 (4.7%)	0.566
Previous PCI	68 (7.4%)	18 (8.5%)	0.568
Previous CABG	4 (0.4%)	1 (0.5%)	1.000
Previous stroke	28 (3.1%)	18 (8.5%)	<0.001
Atrial fibrillation	20 (2.2%)	1 (0.5%)	0.153
Presentation/procedure			
SBP at admission, mmHg	130.0 [112.2–150.0]	130.0 [110.0–146.0]	0.274
DBP at admission, mmHg	80.0 [70.0–90.0]	75.0 [65.8–90.0]	0.001
Heart rate at admission, bpm	75.0 [66.0–85.0]	70.0 [60.0–84.0]	0.002
LVEF, %	55.5 [50.0–60.0]	53.0 [45.8–60.0]	<0.001
Complex PCI	129 (14.1%)	28 (13.2%)	0.826
Multivessel PCI	92 (10.0%)	22 (10.4%)	0.899
Left main PCI	8 (0.9%)	2 (0.9%)	1.000
PCI failure	5 (0.6%)	1 (0.5%)	1.000
Kidney function			
Baseline creatinine, mg/dL	0.9 [0.8–1.0]	0.9 [0.8–1.0]	0.650
Baseline eGFR (MDRD), mL/min/1.73 m^2^	92.0 [79.5–105.4]	75.4 [68.0–87.1]	<0.001
3–6-month creatinine, mg/dL	0.9 [0.8–1.0]	0.9 [0.8–1.1]	<0.001
3–6-month eGFR (MDRD), mL/min/1.73 m^2^	93.0 [81.4–105.3]	71.1 [61.8–81.9]	<0.001
Laboratory findings			
Total cholesterol, mg/dL	195.0 [164.0–222.0]	181.5 [149.0–215.0]	0.002
Triglyceride, mg/dL	150.0 [104.0–217.0]	115.0 [83.0–158.2]	<0.001
HDL-C, mg/dL	41.0 [34.0–50.0]	44.0 [37.0–55.0]	<0.001
LDL-C, mg/dL	130.0 [101.0–154.0]	118.0 [91.0–149.0]	0.009
NT-proBNP, pg/mL	130.3 [54.6–355.5]	584.6 [239.0–1,677.0]	<0.001
HbA1c, %	5.9 [5.6–6.5]	5.9 [5.6–6.4]	0.976
WBC, ×10^3^/µL	10.3 [8.2–12.6]	9.4 [7.6–11.7]	0.001
Hemoglobin, g/dL	14.6 [13.6–15.5]	12.7 [11.6–13.6]	<0.001
Platelet, ×10^3^/µL	243.0 [205.0–283.0]	246.0 [200.8–295.2]	0.360
Discharge medications			
Beta-blocker	744 (81.1%)	164 (77.4%)	0.213
ACE inhibitor	120 (13.1%)	24 (11.3%)	0.568
ARB	631 (68.8%)	150 (70.8%)	0.621
Lipid-lowering therapy	879 (95.9%)	204 (96.2%)	1.000
Aspirin	903 (98.5%)	209 (98.6%)	1.000
Clopidogrel	674 (73.5%)	179 (84.4%)	<0.001
Prasugrel	42 (4.6%)	4 (1.9%)	0.083
Ticagrelor	175 (19.1%)	24 (11.3%)	0.007

No incident CKD combines patients with no AKI and transient AKI. Values are presented as median (interquartile range) or number (percentage). Comparisons used the Mann–Whitney U test for continuous variables and the chi-square or Fisher exact test for categorical variables, as appropriate. Registry-defined incident CKD was based on the prespecified Cockcroft-Gault creatinine-clearance threshold at the 3–6-month assessment.

**Figure 2:**
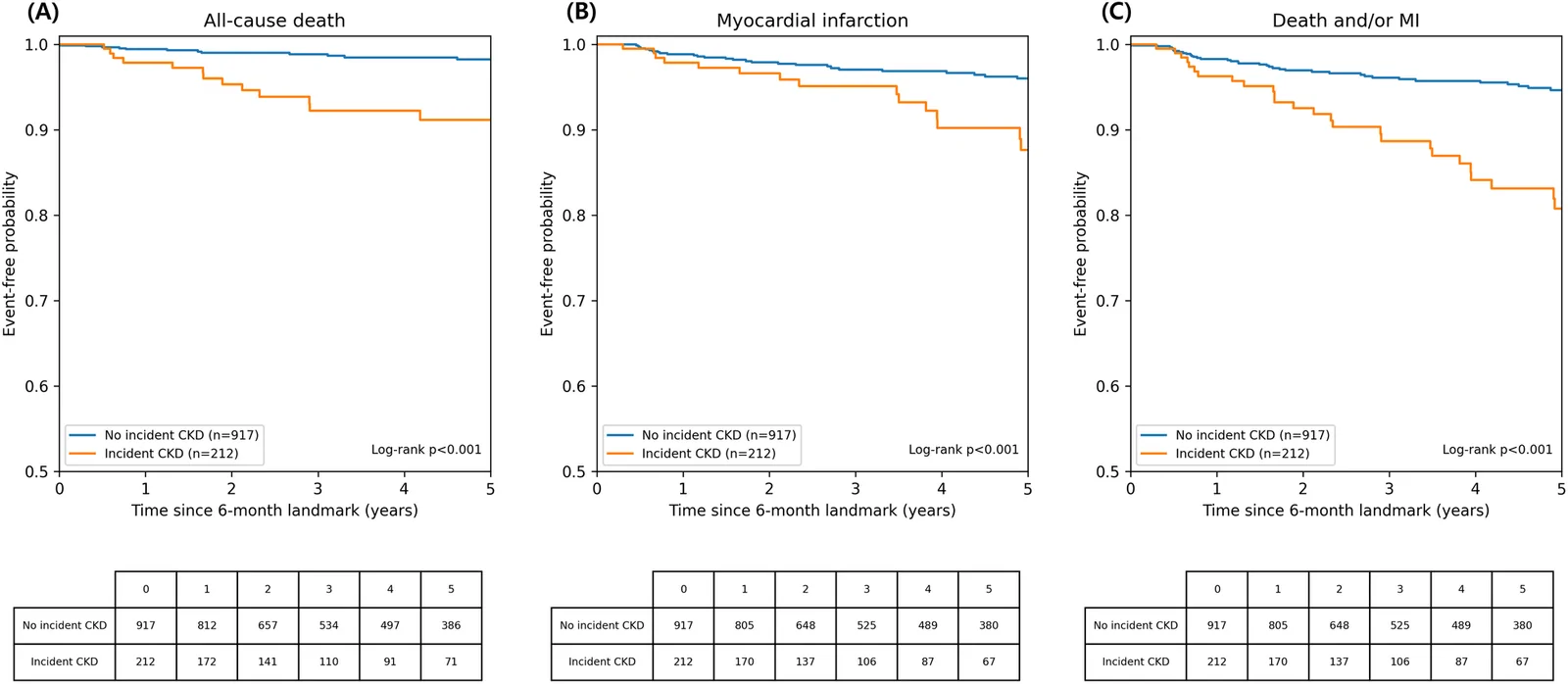
Kaplan–meier event-free survival curves according to incident CKD status in the 6-month landmark cohort. **(A)** All-cause death. **(B)** Myocardial infarction. **(C)** Death and/or myocardial infarction. Blue lines indicate patients without incident CKD (*n* = 917), and orange lines indicate patients with incident CKD (*n* = 212). Follow-up time was calculated from the 6-month landmark. Numbers at risk are shown below each panel at yearly intervals. *P* values were calculated using the log-rank test. CKD, chronic kidney disease; MI, myocardial infarction.

All-cause death occurred in 15/917 (1.6%) patients without incident CKD and 18/212 (8.5%) patients with incident CKD. MI occurred in 32/917 (3.5%) and 19/212 (9.0%), respectively, while death and/or MI occurred in 45/917 (4.9%) and 35/212 (16.5%), respectively. In multivariable Cox analysis, registry-defined incident CKD was associated with all-cause death (multivariable Cox HR 3.44, 95% CI 1.18–10.06; *P* = 0.024), MI (multivariable Cox HR 3.75, 95% CI 1.59–8.87; *P* = 0.003), and death and/or MI (multivariable Cox HR 3.61, 95% CI 1.83–7.14; *P* < 0.001) (Table 4).

**Table 4:** Cox proportional hazards analyses according to incident CKD status.

Outcome	No incident CKD, n/N (%)	Incident CKD, n/N (%)	Unmultivariable Cox HR (95% CI)	*P* value	Multivariable Cox HR (95% CI)	*P* value
All-cause death	15/917 (1.6%)	18/212 (8.5%)	6.81 (3.39–13.68)	<0.001	3.44 (1.18–10.06)	0.024
Myocardial infarction	32/917 (3.5%)	19/212 (9.0%)	3.12 (1.77–5.53)	<0.001	3.75 (1.59–8.87)	0.003
Death and/or MI	45/917 (4.9%)	35/212 (16.5%)	4.24 (2.72–6.63)	<0.001	3.61 (1.83–7.14)	<0.001

Reference group: no incident CKD. Cox follow-up began at the 6-month landmark. Adjusted models included age, sex, diabetes mellitus, LVEF, and baseline MDRD eGFR.

## Discussion

In this multicenter registry study of STEMI patients treated with primary PCI, kidney recovery trajectory was associated with subsequent prognosis. The principal finding was that registry-defined incident CKD, reflecting kidney dysfunction still present at the 3–6-month assessment after in-hospital AKI, identified a markedly high-risk group. In contrast, transient AKI with subsequent recovery was not independently associated with death or MI after multivariable adjustment. The findings were consistent in both the three-trajectory and two-group analyses and were evaluated from a 6-month landmark so that kidney trajectory was established before outcome follow-up began.

Our results extend previous observations concerning kidney recovery after STEMI. Kofman et al. reported that prolonged kidney dysfunction after AKI in patients treated with primary PCI was associated with worse long-term survival and adverse kidney outcomes (7). Itach et al. showed that delayed or absent AKI recovery after STEMI was associated with substantially higher long-term mortality, whereas early recovery carried a more favorable prognosis (8). Frydman et al. further demonstrated that acute kidney disease persisting for 7–90 days after STEMI-associated AKI was common and was associated with higher 1-year mortality (9). The present study adds to this literature by examining an operational CKD phenotype at the later 3–6-month assessment, beyond the conventional acute kidney disease period, and by separating no AKI, transient AKI, and persistent post-AKI dysfunction in a multicenter cohort.

Several mechanisms may account for the observed association. Persistent kidney dysfunction may reflect a more severe systemic insult during the index STEMI, incomplete recovery from kidney ischemia, maladaptive tubular repair, chronic inflammation, and progressive nephron loss (5, 6, 13, 14). Contemporary prospective data in patients with STEMI undergoing primary PCI also indicate that AKI remains a clinically relevant complication in STEMI, arising from multifactorial insults including hemodynamic instability, systemic inflammation, and iodinated contrast exposure (15). In addition, recent imaging data in acute myocardial infarction demonstrate that ventricular-arterial coupling is closely related to left ventricular systolic and diastolic performance, underscoring the importance of the hemodynamic milieu after myocardial infarction (16). Once chronic kidney dysfunction is established, endothelial dysfunction, oxidative stress, neurohormonal activation, vascular calcification, and accelerated atherosclerosis may further increase the risk of recurrent ischemic events and death (17, 18). The close interaction between chronic kidney disease and cardiac dysfunction, including shared neurohormonal pathways, has also been emphasized in contemporary cardiovascular imaging literature (19). These mechanisms provide a biologically plausible cardiorenal framework for the observed association, although they were not directly tested in the present study.

The three-group analysis provides clinically useful context. Although transient AKI was associated with numerically higher crude event rates, it was not independently associated with death, MI, or death and/or MI after adjustment. In contrast, registry-defined incident CKD was associated with approximately fourfold higher adjusted hazards for these outcomes compared with no AKI. These findings suggest that the occurrence of in-hospital AKI alone does not fully characterize subsequent risk and that the kidney recovery trajectory observed at follow-up may provide additional prognostic information. Because the transient-AKI subgroup was small and had few post-landmark events, direct transient-AKI versus incident-CKD effect estimates would be imprecise and were not emphasized.

The hemodynamic and functional nature of transient AKI in STEMI may partly explain its more favorable prognosis. AKI occurring in the setting of acute hemodynamic compromise—characterized by reduced cardiac output, hypotension, and volume depletion during the index event—may predominantly reflect prerenal, reversible decrements in glomerular filtration that resolve as hemodynamics are restored with revascularization and supportive care. Supporting this interpretation, patients with transient AKI in our cohort showed numerically lower admission systolic blood pressure compared with the no-AKI group (Table 1), consistent with a hemodynamically driven insult. Once cardiac function stabilizes and renal perfusion recovers, structural nephron injury may be limited, allowing kidney function to return toward baseline. By contrast, persistent post-AKI kidney dysfunction may reflect irreversible structural injury, more advanced underlying parenchymal disease, or ongoing cardiorenal interactions that impair recovery. This distinction between hemodynamic and structural AKI subtypes has clinical implications: while transient AKI warrants vigilance during the acute phase, patients who achieve kidney recovery by the 3–6-month assessment appear to carry a prognosis more similar to those without AKI, at least over the post-landmark follow-up period examined here.

From a clinical perspective, the findings support structured kidney reassessment after STEMI-associated AKI. Patients with persistent kidney impairment should undergo comprehensive CKD evaluation, including repeat measurement of serum creatinine/eGFR and urine albumin-to-creatinine ratio when available, with classification according to both GFR and albuminuria categories. Cardiovascular and kidney risk-reduction strategies should include optimization of blood pressure, glycemic control, intensive lipid-lowering therapy, evidence-based renin-angiotensin system blockade where indicated, and sodium-glucose cotransporter-2 inhibitor therapy in patients who meet contemporary indications. Recent Korean real-world data after acute coronary syndrome further highlight the continuing importance of optimizing lipid-lowering treatment and secondary prevention (20). The present observational study does not establish that these interventions specifically modify the excess risk associated with registry-defined incident CKD, but it identifies a population in whom intensified surveillance and guideline-directed management are particularly relevant.

### Study limitations

Several limitations should be considered. First, this was an observational registry analysis, and residual confounding cannot be excluded despite multivariable adjustment. Second, kidney trajectory was classified using the kidney function measurement available within a 3–6-month window rather than at an identical prespecified day, and serial post-discharge measurements sufficient to demonstrate chronicity by contemporary KDIGO criteria were not systematically available for every patient. Accordingly, the term registry-defined incident CKD denotes an operational phenotype of persistent post-AKI kidney dysfunction compatible with CKD rather than definitive KDIGO CKD phenotyping in every patient. Within the KDIGO AKI–AKD–CKD continuum, the intermediate acute kidney disease (AKD) phase—defined as kidney dysfunction persisting for 7 to 90 days after the initial insult—was not systematically captured in this registry. The 3–6-month assessment reflects the entry into the CKD window (persistence ≥90 days) rather than a full characterization of the AKD-to-CKD transition. Furthermore, the classification relied on a single kidney function measurement rather than the two or more measurements at least 90 days apart required by contemporary KDIGO criteria to confirm CKD; accordingly, some patients classified as incident CKD may represent incomplete characterization of recovery, and some classified as transient AKI may represent delayed recovery beyond the assessment window.

Third, the original registry kidney classification used Cockcroft-Gault-estimated creatinine clearance, which is not equivalent to body-surface-area-normalized eGFR and is not the preferred contemporary method for CKD staging; contemporary equations such as CKD-EPI are generally preferred. MDRD eGFR is reported descriptively, but the original registry classification was retained to preserve consistency with the prespecified dataset. The equation used may have influenced classification, particularly among patients close to the 60 mL/min threshold. Fourth, albuminuria and urine albumin-to-creatinine ratio were not systematically available, so baseline CKD with preserved GFR but albuminuria could not be fully identified.

Fifth, complete KDIGO AKI stage distribution, peak and discharge creatinine, dialysis-requiring AKI, contrast volume, cardiogenic shock/Killip class, mechanical circulatory support, and CABG performed during the index hospitalization were not uniformly available across the analytic registry dataset. These factors may be associated with both persistent kidney dysfunction and clinical outcomes and therefore may contribute to residual confounding. Sixth, although all 1,239 patients included in the kidney-trajectory cohort had a 3–6-month kidney assessment, the study design cannot address patients who would not undergo such follow-up in routine practice. Seventh, the study was conducted at two tertiary centers in South Korea and may not be fully generalizable to other populations.

Eighth, the recruitment period spanned 2010–2020, during which PCI practice and secondary prevention evolved, and residual calendar-time effects, including the early COVID-19 period, cannot be excluded. Ninth, the 6-month landmark design reduces guarantee-time bias but conditions inference on survival and adequate follow-up through the landmark; the reported associations therefore represent post-landmark risks among evaluable survivors rather than risks beginning at the index STEMI. Finally, the proportional hazards assumption was not uniform for every covariate, and the reported Cox HRs should be interpreted as average relative hazards over follow-up.

## Conclusions

Among STEMI patients undergoing primary PCI, registry-defined incident CKD following in-hospital AKI was associated with substantially higher subsequent risks of all-cause death and MI, whereas transient AKI with recovery was not independently associated with these outcomes. A 6-month landmark analysis supports kidney recovery trajectory as a clinically meaningful post-STEMI risk marker and underscores the importance of structured kidney surveillance and cardiovascular risk management after AKI.

## Data Availability

The original contributions presented in the study are included in the article/Supplementary Material, further inquiries can be directed to the corresponding author.
